# The complete chloroplast genome of *Erodium stephanianum* (Geraniaceae)

**DOI:** 10.1080/23802359.2024.2419962

**Published:** 2024-11-12

**Authors:** Xinyu Gao, Mingqiang Lv, Yuning Xie, Wei Shi

**Affiliations:** a Shandong University of Traditional Chinese Medicine, Jinan, China; b Jinan Integrated Traditional Chinese and Western Medicine Hospital, Jinan, China; c School of Public Health, North China University of Science and Technology, Tangshan, China

**Keywords:** Chloroplast genome, erodium stephanianum, phylogenetic analyses

## Abstract

*Erodium stephanianum* (*Erodium stephanianum* Willd. Sp. Pl. 1800) has not had its complete chloroplast genome reported, which limits our understanding of its genetics and evolution. In this study, we assembled and annotated its chloroplast genome, revealing a quadripartite structure with 76 protein-coding genes. Repeat analysis indicated the presence of simple sequence repeats. Phylogenetic analysis confirmed *E. stephanianum's* placement within the genus Erodium of the Geraniaceae family. These findings offer valuable genomic resources for comparative studies in Erodium and Geraniaceae, aiding genetic diversity and phylogenetic analyses.

## 1. Introduction

*Erodium stephanianum* (*E. stephanianum*) is a perennial herbaceous species commonly used in traditional Chinese medicine. It possesses medicinal properties for various ailments including rheumatism, meridian cleansing, blood circulation enhancement, heat and toxin clearance, pathogen and spoilage bacteria inhibition, as well as diarrhea and dysentery relief (Han et al. 2023; Kong et al. 2023; Zhang et al. 1995). Chloroplast genomes provide insights into the evolutionary relationships among species, contribute to understanding phylogenetic frameworks (Raman et al. 2023; Xu et al. 2024). In this study, we successfully assembled the CP genome of *E. stephanianum* and elucidated its phylogenetic position within the Erodium genus. By reporting the first complete plastome of *E. stephanianum*, we aim to provide a foundational genomic resource that will facilitate future comparative genomic and genetic studies within both the *Erodium* genus and the *Geraniaceae* family.

## 2. Materials and methods

**Figure 1.**
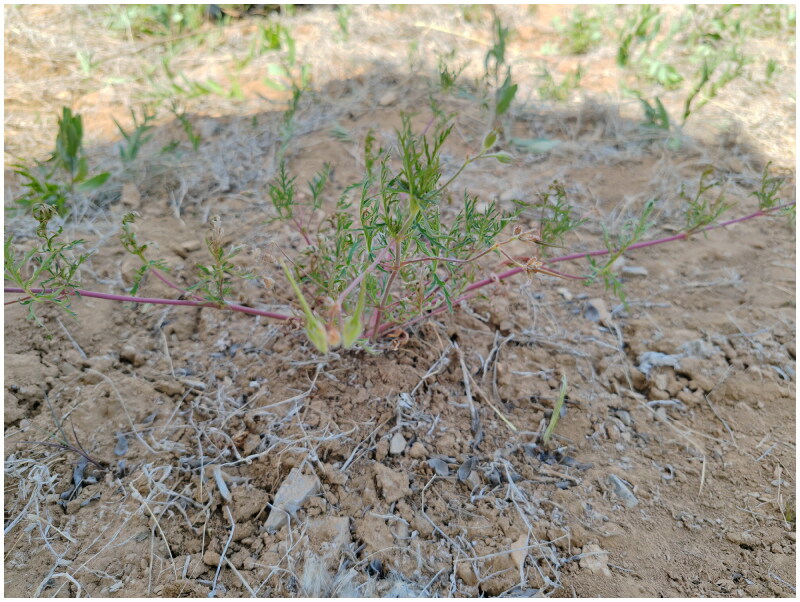
The reference image of *E. stephanianum*. This photo was taken by the author of this article, Xinyu Gao and Yuning Xie.

In June 2023, live *E. stephanianum* leaves were collected from Moyun Mountain, Jinan City, Shandong Province (36°20′31.0308″ N, 117°54′43.4772″ E). *E. stephanianum* was not an endangered or protected species and specific permission for the collection of *E. stephanianum* was not required. A specimen was deposited at the herbarium of the College of Life Science, North China University of Science and Technology (Yuning Xie, xyn0634@gmail.com) under the voucher number NCST20230623002 (Figure 1).

The samples were rinsed, cleaned with DEPC water, and stored at −80 °C until use. Total DNA was extracted following the protocol of the TIANamp Genomic DNA Extraction Kit (TIANamp Genoic DNA Kit) from the Tiangen company. The extracted DNA was then fragmented to construct an Illumina paired-end library and sequenced using the Illumina NovaSeq 6000 platform (Illumina, Inc; San Diego, CA, USA). We obtained 9.8 Gb high quality pair-end reads for *E. stephanianum*. De novo genome assembly was performed using GetOrganelle (v.1.7.5) (Jin et al. 2020) with default parameters. The chloroplast genome was annotated using CPGAVAS2 (Shi et al. 2019) with a reference genome (GenBank accession number: NC_025906.1), and OGDRAW (Greiner et al. 2019) was employed to visualize the chloroplast genome map. The tRNAs of the chloroplast genome were annotated using tRNAscan-SE, and the rRNA was annotated using BLASTn (Chen et al. 2015). Any annotation errors in each chloroplast genome were manually corrected using CPGView (Liu et al. 2023) and Apollo (Lewis et al. 2002).

Closely related species of *E. stephanianum* were selected based on their genetic relationship. Complete mitogenome sequences of these species were downloaded from NCBI. The shared chloroplast genes of these species were extracted using PhyloSuite (Zhang et al. 2020) software. Multiple sequence alignment analysis was performed using MAFFT with a bootstrap value of 1000 (Katoh et al. 2002; Katoh et al. 2019), and phylogenetic analysis was conducted using IQ-TREE based on coding sequences (Supplement Data) (Minh et al. 2020). The results of the phylogenetic analysis were visualized using iTOL software (Letunic and Bork 2021).

## 3. Results

**Figure 2.**
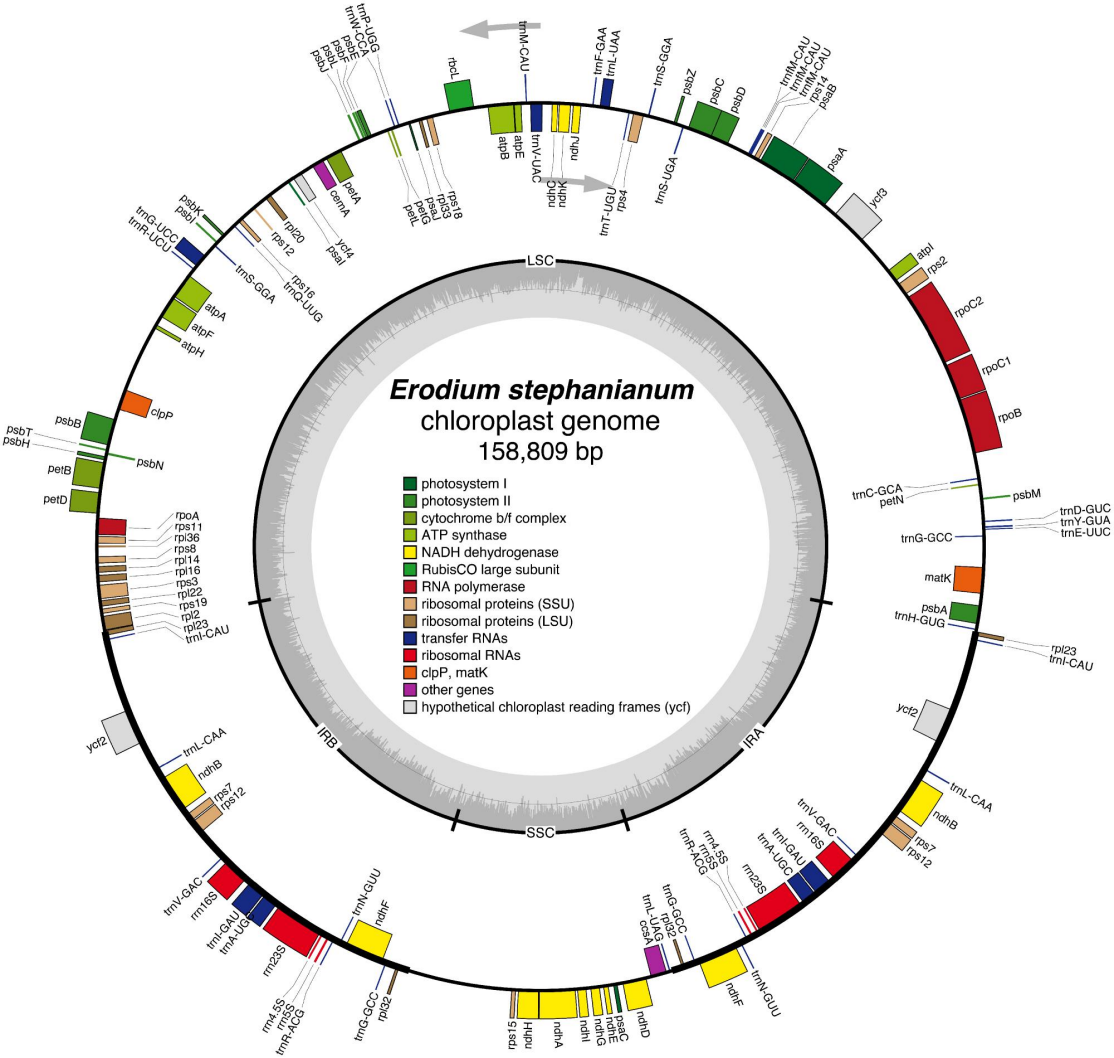
The chloroplast genome map of *E. stephanianum*. Genes on the inside of the circle were transcribed in a clockwise direction and genes on the outside of the circle were transcribed in a counterclockwise direction.

The complete chloroplast genome of *E. stephanianum* (PP234476.1) was 158,809 bp in length and depth for average, maximal and minimal were 3994.55x, 7403x and 212x (Supplementary Figure 1). Meanwhile, the structure of the trans-spliced gene shown in Supplementary Figure 2 and trans- splicing gene shown in Supplementary Figure 3. It consisted of a large single copy (LSC) region spanning 89,129 bp, a small single copy (SSC) region spanning 15,194 bp, and a pair of inverted repeats (IR) regions spanning 27,243 bp (Figure 2). The overall GC content of the *E. stephanianum* chloroplast genome was 40.06%, higher than that of the LSC (38.58%) and SSC (36.05%), but lower than that of the IRs (43.62%) (Table S1). The chloroplast genome of *E. stephanianum* encoded 76 unique protein-coding genes, 27 tRNA genes, and 4 rRNA genes. These protein-coding genes comprised 14 gene families, including 11 NADH dehydrogenase subunit genes, 5 photosystem I subunit genes, 16 photosystem II subunit genes, 6 cytochrome b/f complex subunit genes, 6 ATP synthase subunit genes, 1 ribulose-1,5-bisphosphate carboxylase/oxygenase large subunit gene, 4 DNA-dependent RNA polymerase genes, 9 ribosomal large subunit genes, 12 ribosomal small subunit genes, 1 mature enzyme gene, 1 c-type cytochrome synthase gene, 1 membrane protein gene, 1 protease gene, 1 acetyl-CoA-carboxylase subunit gene, 1 translation initiation factor gene, and 3 conserved open reading frame genes (Table S2).

**Figure 3.**
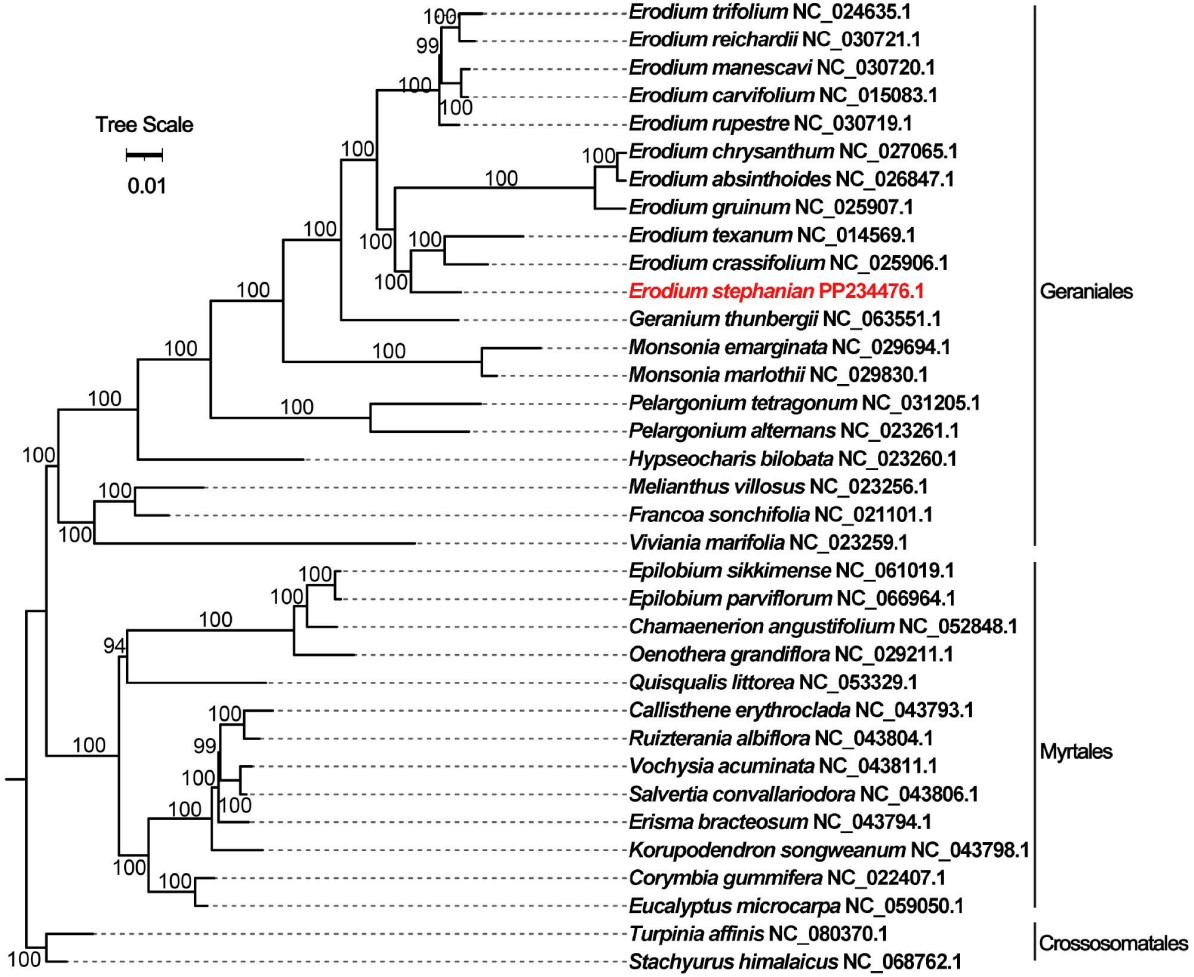
The phylogenetic tree is based on chloroplast genome sequences of *E. stephanianum* species from the Geraniaceae family. The following sequences were used: *Erodium absinthoides* NC_026847.1 (Kong et al. 2023), *Erodium carvifolium* NC_015083.1 (Chris Blazier et al. 2011), *Erodium chrysanthum* NC_027065.1, *Erodium crassifolium* NC_025906.1 (Cohen et al. 2020), *Erodium gruinum* NC_025907.1 (Al-Hadid et al. 2019), *Erodium manescavi* NC_030720.1 (Fecka et al. 2001), *Erodium reichardii* NC_030721.1, *Erodium rupestre* NC_030719.1, *Erodium texanum* NC_014569.1 (Guisinger et al. 2011), *Erodium trifolium* NC_024635.1, *Francoa sonchifolia* NC_021101.1 (Weng et al. 2014), *Geranium thunbergia* NC_063551.1 (Schultz et al. 2000), *Hypseocharis bilobate* NC_023260.1, *Melianthus villosus* NC_023256.1 (Weng et al. 2014), *Monsonia emarginata* NC_029694.1 (Ruhlman et al. 2017), *Monsonia marlothii* NC_029830.1 (Semenya and Maroyi 2012), *Pelargonium alternans* NC_023261.1 (Weng et al. 2014), *Pelargonium tetragonum* NC_031205.1, *Viviania marifolia* NC_023259.1 (Weng et al. 2014), *Vochysia acuminata* NC_043811.1, *Salvertia convallariodora* NC_043806.1 (De Mesquita et al. 2017), *Oenothera grandiflora* NC_029211.1 (Levy et al. 1975), *Ruizterania albiflora* NC_043804.1, *Quisqualis littorea* NC_053329.1, *Callisthene erythroclada* NC_043793.1, *Epilobium parviflorum* NC_066964.1 (Bratu et al. 2023), *Chamaenerion angustifolium* NC_052848.1 (Efimenko et al. 2021), *Korupodendron songweanum* NC_043798.1 (Droissart et al. 2014), *Epilobium sikkimense* NC_061019.1, *Corymbia gummifera* NC_022407.1, *Erisma bracteosum* NC_043794.1, *Eucalyptus macrocarpa* NC_059050.1 (Poinern et al. 2011), *Stachyurus himalaicus* NC_068762.1 (Wang et al. 2010), *Turpinia affinis* NC_080370.1.

A maximum-likelihood phylogenetic tree was constructed for *E. stephanianum*, incorporating 35 species from three orders of angiosperms. Our phylogenetic indicated strong confidence in the nodes (Figure 3). The chloroplast genome sequences of the plant species are detailed in Table S3. *E. stephanianum*, *Erodium texanum*, and *Erodium crassifolium* were grouped together within the order Geraniales, family Geraniaceae.

## 4. Discussion and conclusion

We present the first annotated chloroplast genome of *E. stephanianum*, describing its structure. The genome has a total length of 158,809 bp and contains 76 annotated protein-coding genes. Phylogenetic analysis confirms that *E. stephanianum* belongs to the *Erodium* genus in the family *Geraniaceae. The erodium* genus is a relevant source of compounds with antioxidant, antimicrobial, and biological activity (Munekata et al. 2019) and main compositions are tannins, flavones, organic acids and volatile oil (Kong et al. 2023). As an important traditional Chinese medicine, *E. stephanianum* should be further explored to promote the development of new drugs and therapeutics for various diseases.

## Supplementary Material

es_Supplementary tables.docx

Supplementary figure3.pdf

Supplementary figure2.pdf

Supplementary figure1.jpg

v4_es_Manuscript_clean copy.docx

es_Supplementary figures.docx

## Data Availability

The genome sequence data that support the findings of this study are openly available in GenBank of NCBI at https://www.ncbi.nlm.nih.gov/ under accession no. PP234476.1. The associated BioProject, SRA, and Bio-Sample numbers are PRJNA1073844, SRR27999911 and SAMN39839336, respectively.
