# Supplementary material for: The complete chloroplast genome of Erodium stephanianum (Geraniaceae)
Source: Mitochondrial DNA B Resour. 2024 Nov 12;9(11):1501–5. doi: 10.1080/23802359.2024.2419962 (PMC11559019; doi:10.1080/23802359.2024.2419962)
Supplement: es_Supplementary tables.docx [file TMDN_A_2419962_SM3113.docx]

**Supplementary Tables**

**Supplementary Table1.** Summary of *E. stephanianum* chloroplast genome structure.

| Region name | Start | End | Length | GC content |
| --- | --- | --- | --- | --- |
| LSC | 1 | 89,129 | 89,129 bp | 38.58% |
| IR | 89,130 | 116,372 | 27,243 bp | 43.62% |
| SSC | 116,373 | 131,566 | 15,194 bp | 36.05% |
| IR | 131,567 | 158,809 | 27,243 bp | 43.62% |

**Supplementary Table2.** Gene composition in the chloroplast of *E. stephanianum*.

| Group of genes | Name of genes |
| --- | --- |
| Subunits of NADH-dehydrogenase | *ndhA ， ndhB(×2) ， ndhC ， ndhD ， ndhE ， ndhF(×2)，ndhG，ndhH，ndhI，ndhJ，ndhK* |
| Subunits of photosystem I | *psaA，psaB，psaC，psaI，psaJ* |
| Subunits of photosystem II | *psbA，psbB，psbC，psbD，psbE，psbF，psbH， psbI，psbJ，psbK，psbL, psbM，psbN，psbT， psbZ，ycf3* |
| Subunits of cytochrome b/f complex | *petA，petB，petD，petG，petL，petN* |
| Subunits of ATP synthase | *atpA，atpB，atpE，atpF，atpH，atpI* |
| Large subunit of rubisco | *rbcL* |
| Small subunit of ribosome | *rps2，rps3，rps4，rps7(×2)，rps8，rps11， rps12(×2)，rps14，rps15，rps16，rps18, rps19* |
| Large subunit of ribosome | *rpl2，rpl14，rpl16，rpl20，rpl22，rpl23(×2)， rpl32(×2)，rpl33，rpl36* |
| DNA dependent RNA polymerase | *rpoA，rpoB，rpoC1，rpoC2* |
| rRNA genes | *rrn4.5S(×2) ， rrn5S(×2) ， rrn16S(×2) ， rrn23S(×2)* |
| tRNA genes | *trnA-UGC(×2)，trnC-GCA，trnD-GUC，trnE- UUC ， trnF-GAA ， trnfM-CAU(×3) ， trnG- GCC(×3) ， trnG-UCC ， trnH-GUG ， trnI- CAU(×2)，trnI-GAU(×2)，trnL-CAA(×2)，trnL- UAA，trnL-UAG，trnM-CAU，trnN-GUU(×2)，trnP-UGG，trnQ-UUG，trnR-ACG(×2)，trnR- UCU，trnS-GGA(×2)，trnS-UGA，trnT-UGU， trnV-GAC(×2)，trnV-UAC，trnW-CCA，trnY- GUA* |
| Maturase | *matK* |
| c-type cytochrom synthesis gene | *ccsA* |
| Envelope membrane protein | *cemA* |
| Protease | *clpP* |
| Genes of unknown functions Open Reading | *cf2(×2)，ycf4* |

**Supplementary Table3.** Evolutionary tree species information.

|  |  | ID |
| --- | --- | --- |
| Geraniales | Erodium stephanianum |  |
|  | Erodium absinthoides chloroplast, complete genome | NC_026847.1 |
|  | Erodium carvifolium chloroplast, complete genome | NC_015083.1 |
|  | Erodium chrysanthum chloroplast, complete genome | NC_027065.1 |
|  | Erodium crassifolium plastid, complete genome | NC_025906.1 |
|  | Erodium gruinum plastid, complete genome | NC_025907.1 |
|  | Erodium manescavi plastid, complete genome | NC_030720.1 |
|  | Erodium reichardii plastid, complete genome | NC_030721.1 |
|  | Erodium rupestre plastid, complete genome | NC_030719.1 |
|  | Erodium texanum plastid, complete genome | NC_014569.1 |
|  | Erodium trifolium plastid, complete genome | NC_024635.1 |
|  | Francoa sonchifolia plastid, complete genome | NC_021101.1 |
|  | Geranium thunbergii voucher TKMII-18-1 chloroplast, complete genome | NC_063551.1 |
|  | Hypseocharis bilobata chloroplast, complete genome | NC_023260.1 |
|  | Melianthus villosus plastid, complete genome | NC_023256.1 |
|  | Monsonia emarginata chloroplast, complete genome | NC_029694.1 |
|  | Monsonia marlothii chloroplast, complete genome | NC_029830.1 |
|  | Pelargonium alternans chloroplast, complete genome | NC_023261.1 |
|  | Pelargonium tetragonum chloroplast, complete genome | NC_031205.1 |
|  | Viviania marifolia chloroplast, complete genome | NC_023259.1 |
| Myrtales | Vochysia acuminata chloroplast, complete genome | NC_043811.1 |
|  | Salvertia convallariodora chloroplast, complete genome | NC_043806.1 |
|  | Oenothera grandiflora strain Stockton 1 chloroplast, complete genome | NC_029211.1 |
|  | Ruizterania albiflora chloroplast, complete genome | NC_043804.1 |
|  | Quisqualis littorea chloroplast, complete genome | NC_053329.1 |
|  | Callisthene erythroclada chloroplast, complete genome | NC_043793.1 |
|  | Epilobium parviflorum voucher JSY2107001 chloroplast, complete genome | NC_066964.1 |
|  | Chamaenerion angustifolium chloroplast, complete genome | NC_052848.1 |
|  | Korupodendron songweanum chloroplast, complete genome | NC_043798.1 |
|  | Epilobium sikkimense chloroplast, complete genome | NC_061019.1 |
|  | Corymbia gummifera chloroplast, complete genome | NC_022407.1 |
|  | Erisma bracteosum chloroplast, complete genome | NC_043794.1 |
|  | Eucalyptus microcarpa chloroplast, complete genome | NC_059050.1 |
| Crossosomatales | Stachyurus himalaicus chloroplast, complete genome | NC_068762.1 |
|  | Turpinia affinis chloroplast, complete genome | NC_080370.1 |
