# Supplementary figures and images for: The complete chloroplast genome of Erodium stephanianum (Geraniaceae)
Source: Mitochondrial DNA B Resour. 2024 Nov 12;9(11):1501–5. doi: 10.1080/23802359.2024.2419962 (PMC11559019; doi:10.1080/23802359.2024.2419962)

# Cis-splicing Genes

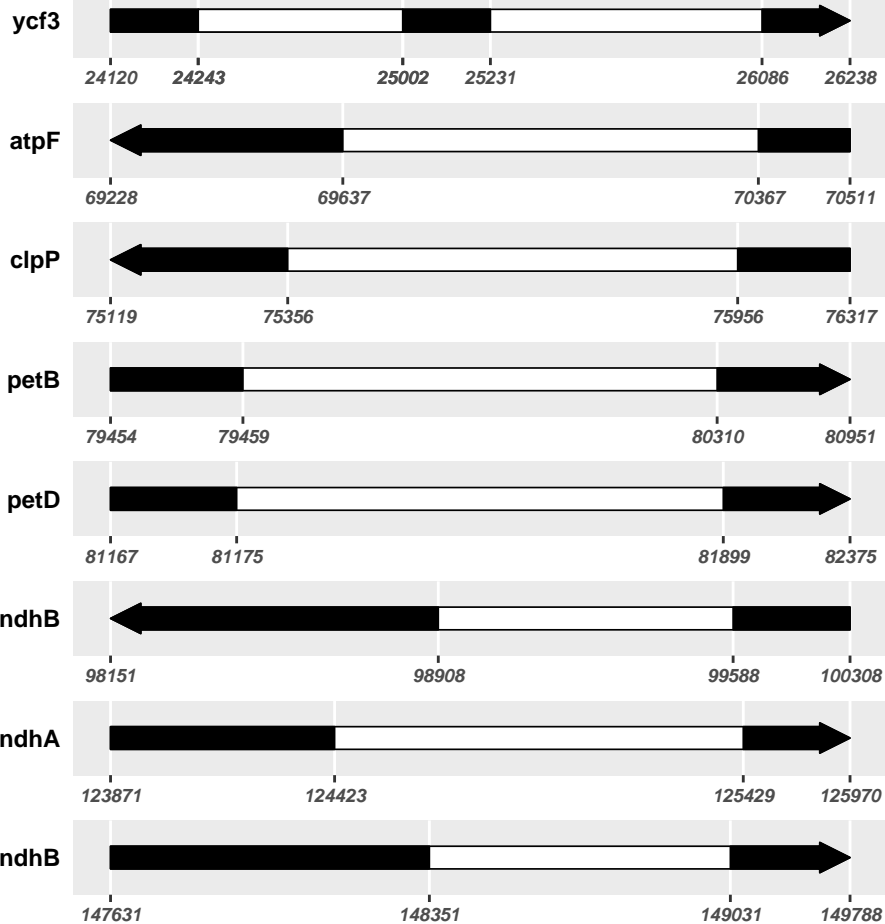

Exon  
Intron

Supplement: Supplementary figure3.pdf [file TMDN_A_2419962_SM3112.pdf]

# Trans-splicing Genes

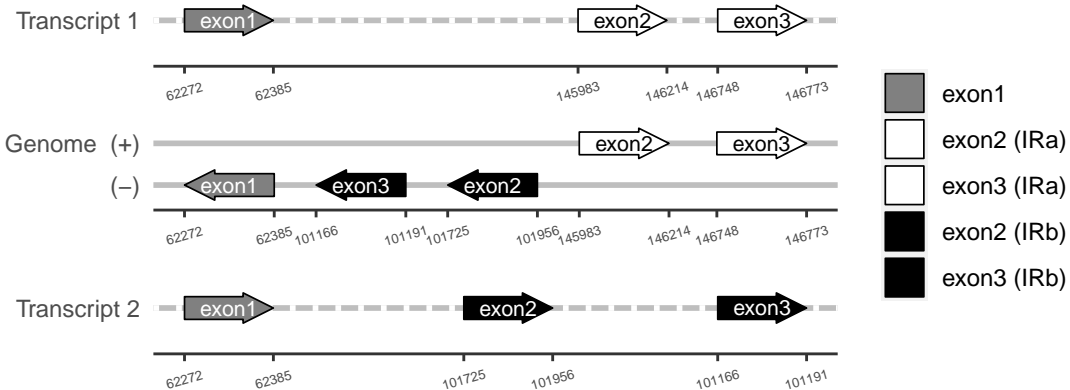

Supplement: Supplementary figure2.pdf [file TMDN_A_2419962_SM3111.pdf]

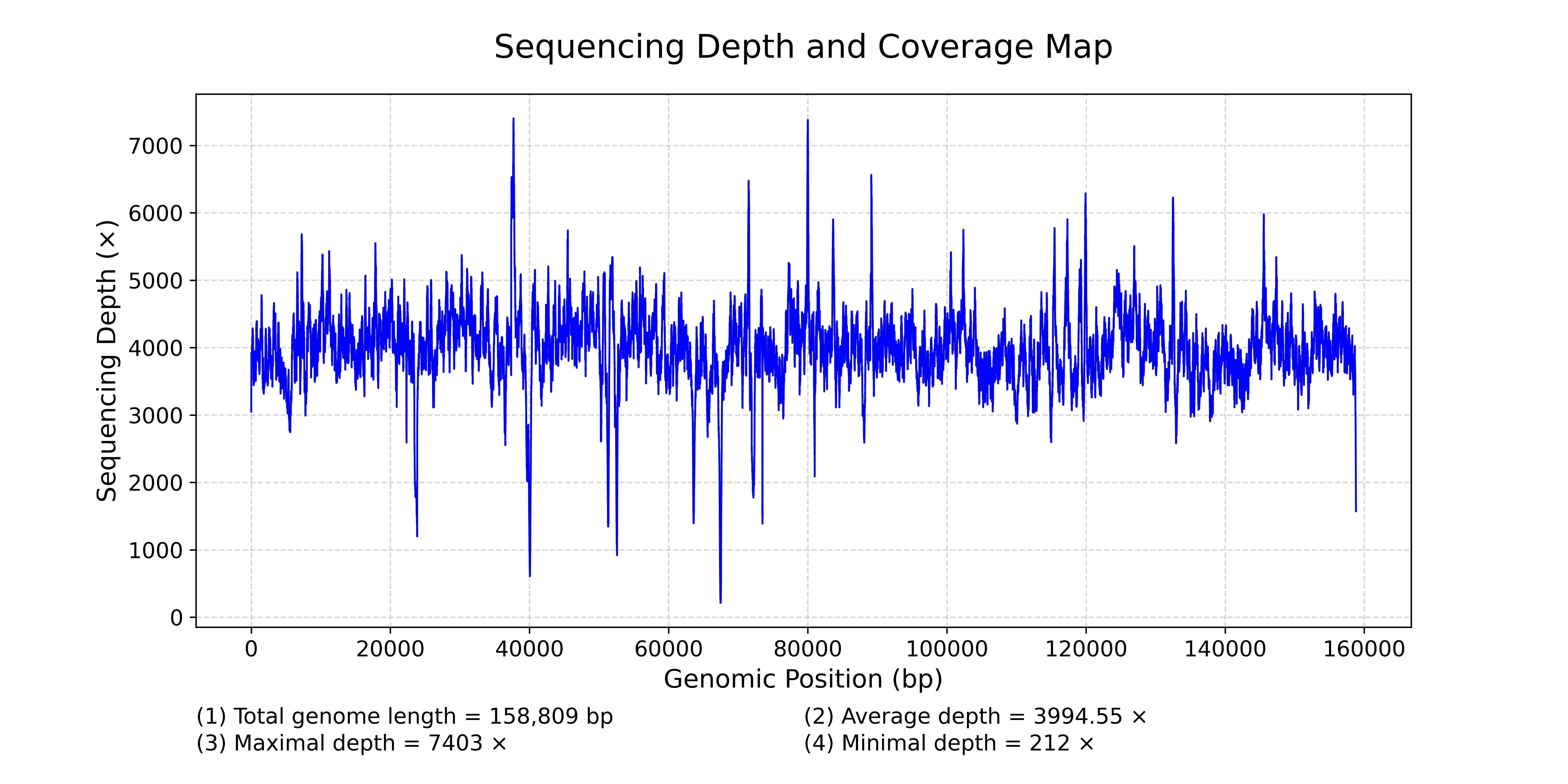

Supplement: Supplementary figure1.jpg [file TMDN_A_2419962_SM3110.jpg]
