# Supplementary material for: The complete chloroplast genome of Erodium stephanianum (Geraniaceae)
Source: Mitochondrial DNA B Resour. 2024 Nov 12;9(11):1501–5. doi: 10.1080/23802359.2024.2419962 (PMC11559019; doi:10.1080/23802359.2024.2419962)
Supplement: v4_es_Manuscript_clean copy.docx [file TMDN_A_2419962_SM3109.docx]

**The Complete Chloroplast Genome of Erodium Stephanianum (Geraniaceae)**

Xinyu Gao^1,†^, Mingqiang Lv^2,†^, Yuning Xie^3^, and Wei Shi^1,^*

^1^ Shandong University of Traditional Chinese Medicine, 250355, Jinan, China

^2^ Jinan Integrated Traditional Chinese and Western Medicine Hospital, 271100, Jinan, China

^3^ School of Public Health, North China University of Science and Technology, 063210, Tangshan, China

^†^ These authors contribute equally to this work.

^*^ Corresponding author: Wei Shi

Email address: w19781214@163.com

Wenyuan East Street No.8, Jinan City, Shandong Province, 250355, China

**Abstract**

*Erodium stephanianum* (*Erodium stephanianum* Willd*.* Sp. Pl. 1800), a perennial herbaceous species commonly used in traditional Chinese medicine, possesses medicinal properties for various ailments. However, the complete chloroplast genome of *E. stephanianum* has not been reported, limiting our understanding of its genomic characteristics and evolutionary history. In this study, we successfully assembled and annotated the chloroplast genome of *E. stephanianum*. The genome exhibits a quadripartite structure and consists of 76 annotated protein-coding genes. Repeat analysis revealed the presence of simple sequence repeats and repeat sequences. Phylogenetic analysis confirmed that *E. stephanianum* belongs to the genus Erodium in the family Geraniaceae. Our findings provide valuable genomic resources for comparative studies within the genus Erodium and Geraniaceae, facilitating genetic diversity analysis and phylogenetic investigations.

**Keywords**

Chloroplast genome; erodium stephanianum; phylogenetic analyses

**1 Introduction**

*Erodium stephanianum* (*E. stephanianum*) is a perennial herbaceous species commonly used in traditional Chinese medicine. It possesses medicinal properties for various ailments including rheumatism, meridian cleansing, blood circulation enhancement, heat and toxin clearance, pathogen and spoilage bacteria inhibition, as well as diarrhea and dysentery relief (Zhang *et al.* 1995; Han *et al.* 2023; Kong *et al.* 2023). Chloroplast genomes provide insights into the evolutionary relationships among species, contribute to understanding phylogenetic frameworks(Raman *et al.* 2023; Xu *et al.* 2024). In this study, we successfully assembled the CP genome of *E. stephanianum* and elucidated its phylogenetic position within the Erodium genus. By reporting the first complete plastome of *E. stephanianum*, we aim to provide a foundational genomic resource that will facilitate future comparative genomic and genetic studies within both the *Erodium* genus and the *Geraniaceae* family.

**2 Materials and Methods**

In June 2023, live *E. stephanianum* leaves were collected from the Moyun Mountain, Jinan City, Shandong Province (36°20'31.0308" N, 117°54'43.4772" E). *E. stephanianum* was not an endangered or protected species and specific permission for the collection of *E. stephanianum* was not required. A specimen was deposited at the herbarium of the College of Life Science, North China University of Science and Technology (Yuning Xie, xyn0634@gmail.com) under the voucher number NCST20230623002 (Figure 1).


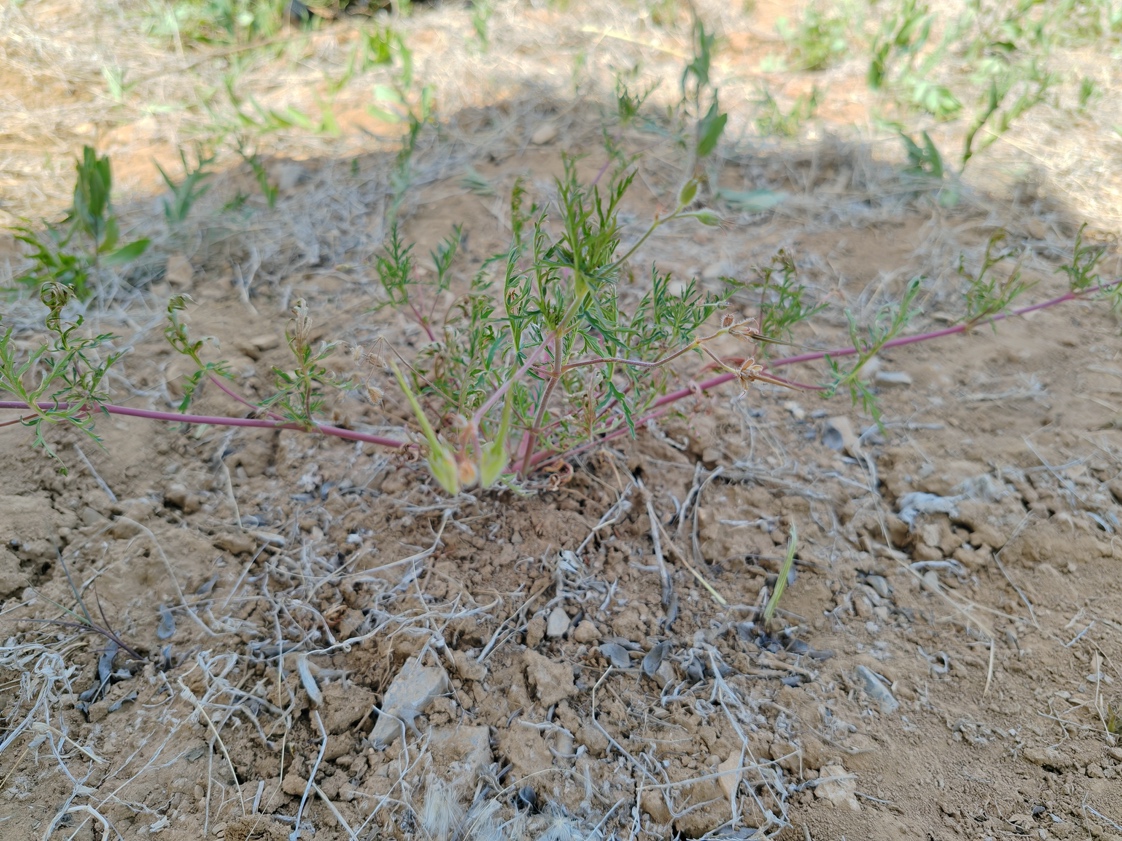


**Figure 1.** The reference image of *E. stephanianum*. This photo was taken by the author of this article, Xinyu Gao and Yuning Xie.

The samples were rinsed, cleaned with DEPC water, and stored at -80°C until use. Total DNA was extracted following the protocol of the TIANamp Genomic DNA Extraction Kit (TIANamp Genoic DNA Kit) from Tiangen company. The extracted DNA was then fragmented to construct an Illumina paired-end library and sequenced using the Illumina NovaSeq 6000 platform (Illumina, Inc; San Diego, CA, USA). We obtained 9.8Gb high quality pair-end reads for *E. stephanianum*. De novo genome assembly was performed using GetOrganelle (v.1.7.5) (Jin *et al.* 2020) with default parameters. The chloroplast genome was annotated using CPGAVAS2 (Shi *et al.* 2019) with a reference genome (GenBank accession number: NC_025906.1), and OGDRAW (Greiner *et al.* 2019) was employed to visualize the chloroplast genome map. The tRNAs of the chloroplast genome were annotated using tRNAscan-SE, and the rRNA was annotated using BLASTn (Chen *et al.* 2015). Any annotation errors in each chloroplast genome were manually corrected using CPGView (Liu *et al.* 2023) and Apollo (Lewis *et al.* 2002).

Closely related species of *E. stephanianum* were selected based on their genetic relationship. Complete mitogenome sequences of these species were downloaded from NCBI. The shared chloroplast genes of these species were extracted using PhyloSuite (Zhang *et al.* 2020) software. Multiple sequence alignment analysis was performed using MAFFT with a bootstrap value of 1000 (Katoh *et al.* 2002; Katoh *et al.* 2019), and phylogenetic analysis was conducted using IQ-TREE based on coding sequences (Supplement Data) (Minh *et al.* 2020). The results of the phylogenetic analysis were visualized using iTOL software (Letunic and Bork 2021).

**3 Results**

The complete chloroplast genome of *E. stephanianum* (PP234476.1) was 158,809 bp in length and and depth for average, maximal and minimal were 3994.55x, 7403x and 212x (Supplementary Figure 1). Meanwhile, the structure of trans-spliced gene shown in Supplementary Figure 2 and trans- splicing gene shown in Supplementary Figure 3. It consisted of a large single copy (LSC) region spanning 89,129 bp, a small single copy (SSC) region spanning 15,194 bp, and a pair of inverted repeats (IR) regions spanning 27,243 bp (Figure 2). The overall GC content of the *E. stephanianum* chloroplast genome was 40.06%, higher than that of the LSC (38.58%) and SSC (36.05%), but lower than that of the IRs (43.62%) (Table S1). The chloroplast genome of *E. stephanianum* encoded 76 unique protein-coding genes, 27 tRNA genes, and 4 rRNA genes. These protein-coding genes comprised 14 gene families, including 11 NADH dehydrogenase subunit genes, 5 photosystem I subunit genes, 16 photosystem II subunit genes, 6 cytochrome b/f complex subunit genes, 6 ATP synthase subunit genes, 1 ribulose-1,5-bisphosphate carboxylase/oxygenase large subunit gene, 4 DNA-dependent RNA polymerase genes, 9 ribosomal large subunit genes, 12 ribosomal small subunit genes, 1 mature enzyme gene, 1 c-type cytochrome synthase gene, 1 membrane protein gene, 1 protease gene, 1 acetyl-CoA-carboxylase subunit gene, 1 translation initiation factor gene, and 3 conserved open reading frame genes (Table S2).


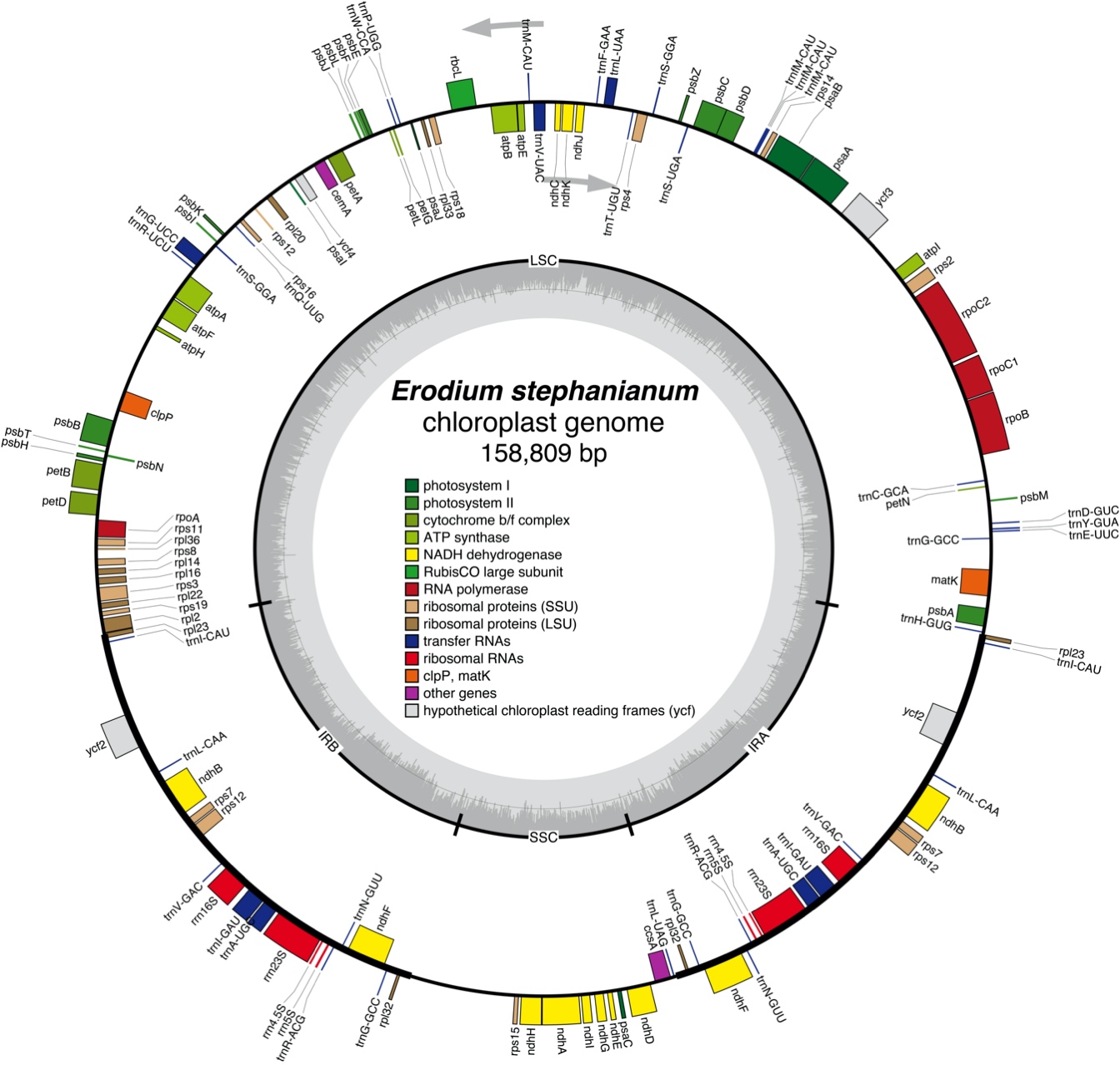


**Figure 2.** The chloroplast genome map of *E. stephanianum*. Genes on the inside of the circle were transcribed in a clockwise direction and genes on the outside of the circle were transcribed in a counterclockwise direction.

A maximum-likelihood phylogenetic tree was constructed for *E. stephanianum*, incorporating 35 species from three orders of angiosperms. Our phylogenetic indicated strong confidence in the nodes (Figure 3). The chloroplast genome sequences of the plant species are detailed in Table S3. *E. stephanianum*, *Erodium texanum*, and *Erodium crassifolium* were grouped together within the order Geraniales, family Geraniaceae.


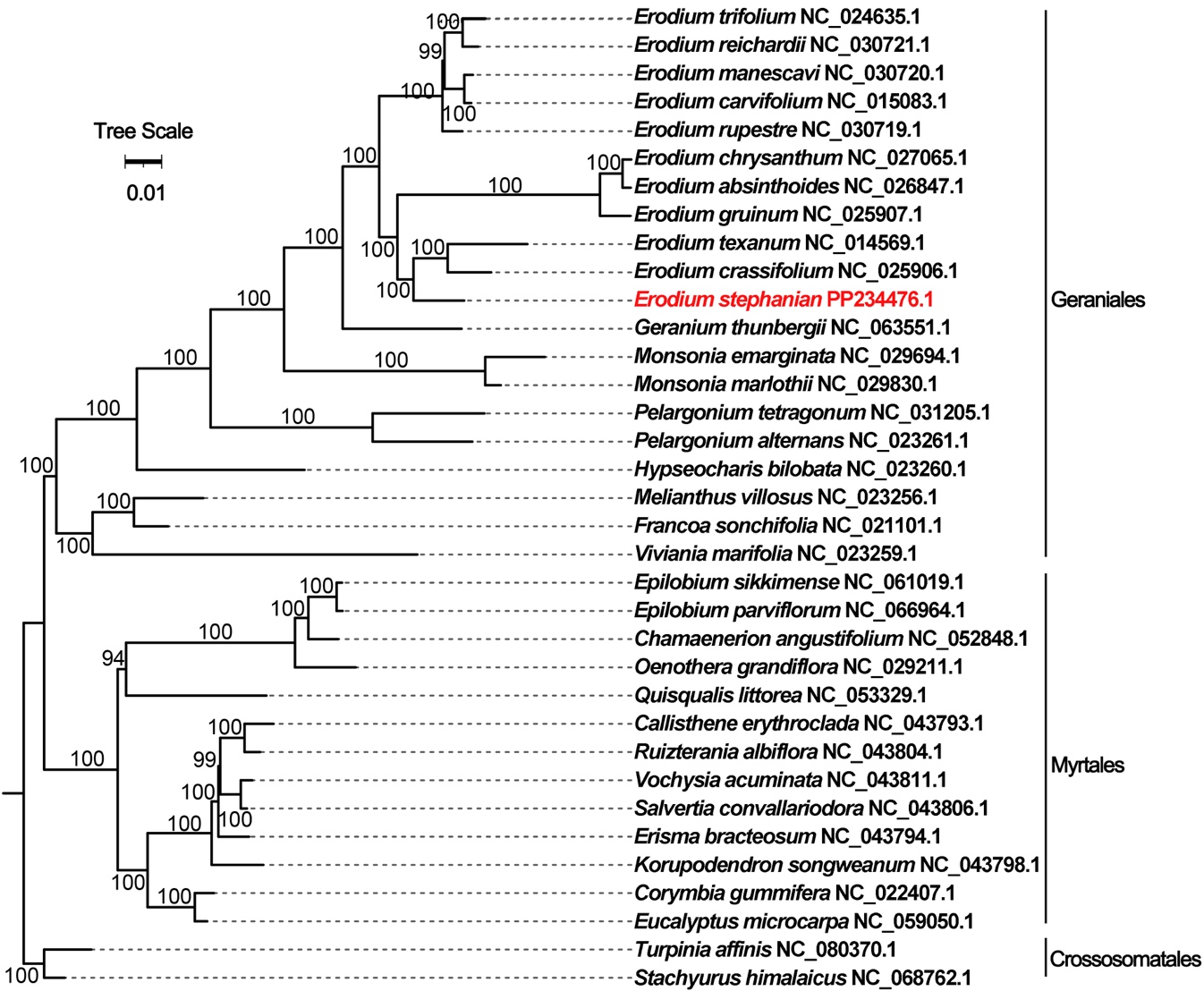


**Figure 3.** The phylogenetic tree is based on chloroplast genome sequences of *E. stephanianum* species from the Geraniaceae family. The following sequences were used: *Erodium absinthoides* NC_026847.1 (Kong *et al.* 2023), *Erodium carvifolium* NC_015083.1 (Chris Blazier *et al.* 2011), *Erodium chrysanthum* NC_027065.1, *Erodium crassifolium* NC_025906.1 (Cohen *et al.* 2020), *Erodium gruinum* NC_025907.1 (Al-Hadid *et al.* 2019), *Erodium manescavi* NC_030720.1 (Fecka *et al.* 2001), *Erodium reichardii* NC_030721.1,

*Erodium rupestre* NC_030719.1, *Erodium texanum* NC_014569.1 (Guisinger *et al.* 2011), *Erodium trifolium* NC_024635.1, *Francoa sonchifolia* NC_021101.1 (Weng *et al.* 2014), *Geranium thunbergia* NC_063551.1 (Schultz *et al.* 2000), *Hypseocharis bilobate* NC_023260.1, *Melianthus villosus* NC_023256.1 (Weng *et al.* 2014), *Monsonia emarginata* NC_029694.1 (Ruhlman *et al.* 2017), *Monsonia marlothii* NC_029830.1 (Semenya and Maroyi 2012), *Pelargonium alternans* NC_023261.1 (Weng *et al.* 2014), *Pelargonium tetragonum* NC_031205.1, *Viviania marifolia* NC_023259.1 (Weng *et al.* 2014), *Vochysia acuminata* NC_043811.1, *Salvertia convallariodora* NC_043806.1 (De Mesquita *et al.* 2017), *Oenothera grandiflora* NC_029211.1 (Levy *et al.* 1975), *Ruizterania albiflora* NC_043804.1, *Quisqualis littorea* NC_053329.1, *Callisthene erythroclada* NC_043793.1, *Epilobium parviflorum* NC_066964.1 (Bratu *et al.* 2023), *Chamaenerion angustifolium* NC_052848.1 (Efimenko *et al.* 2021), *Korupodendron songweanum* NC_043798.1 (Droissart *et al.* 2014), *Epilobium sikkimense* NC_061019.1, *Corymbia gummifera* NC_022407.1, *Erisma bracteosum* NC_043794.1, *Eucalyptus macrocarpa* NC_059050.1 (Poinern *et al.* 2011), *Stachyurus himalaicus* NC_068762.1 (Wang *et al.* 2010), *Turpinia affinis* NC_080370.1.

**4 Discussion and Conclusion**

We present the first annotated chloroplast genome of *E. stephanianum*, describing its structure. The genome has a total length of 158,809 bp and contains 76 annotated protein-coding genes. Phylogenetic analysis confirms that *E. stephanianum* belongs to the *Erodium* genus in the family *Geraniaceae*. *Erodium* genus is a relevant source of compounds with antioxidant, antimicrobial, and biological activity(Munekata *et al.* 2019) and main compositions are tannins, flavones, organic acids and volatile oil(Kong *et al.* 2023). As an important traditional Chinese medicine, *E. stephanianum* should be further explored to promote the development of new drugs and therapeutics for various diseases.

**5 Disclosure statement**

The coauthors do not have any conflict of interest to declare. The authors alone are responsible for the content and composition of the paper.

**6 Ethical approval**

The material involved in the article does not involve ethical conflicts. This species is neither endangered on the CITES catalogue nor collected from a natural reserve, so it did not need specific permissions or licenses. *E. stephanianum* is not endangered plant and was collected in accordance with laws and regulations.

**7 Author contributions**

Xinyu Gao and Wei Shi conceived and supervised the project. Yuning Xie and Xinyu Gao assembled and annotated the cp genome; Mingqiang Lv contributed significantly to phylogenetic analysis and manuscript preparation; Wei Shi was involved in interpretation of the data and revised the manuscript critically for intellectual content. This article was modified and graphed by Yuning Xie and Mingqing Lv. All authors approved the final version to be published and agreed to be accountable for all aspects of the work.

**8 Funding**

This work was supported by Innovation and Development Joint Fund of Natural Science Foundation from Shandong Province [ZR202306250028].

**9 Data availability statement**

The genome sequence data that support the findings of this study are openly available in GenBank of NCBI at https://www.ncbi.nlm.nih.gov/ under the accession no. PP234476.1. The associated BioProject, SRA, and Bio-Sample numbers are

PRJNA1073844, SRR27999911 and SAMN39839336, respectively.

**10 References**

**Al-Hadid KJ, Al-Karablieh N, Sharab A, Mutlak I.** Phytochemical analyses and antibacterial activities of Erodium, Euphorbia, Logoecia and Tamarix species. *J Infect Dev Ctries* 13, 1013-20, 2019

**Bratu MM, Birghila S, Birghila C, Coatu V, Danilov DA, Lupascu N, Vasiliu D, Radu MD.** Correlation Between Toxic Elements and Pesticide Residues in Medicinal Herbs Available in Pharmaceutical Market. *Biol Trace Elem Res* 201, 5848-60, 2023

**Chen Y, Ye W, Zhang Y, Xu Y.** High speed BLASTN: an accelerated MegaBLAST search tool. *Nucleic Acids Res* 43, 7762-8, 2015

**Chris Blazier J, Guisinger MM, Jansen RK.** Recent loss of plastid-encoded ndh genes within Erodium (Geraniaceae). *Plant Mol Biol* 76, 263-72, 2011

**Cohen S, Koltai H, Selvaraj G, Mazuz M, Segoli M, Bustan A, Guy O.** Assessment of the Nutritional and Medicinal Potential of Tubers from Hairy Stork's-Bill (Erodium crassifolium L 'Hér), a Wild Plant Species Inhabiting Arid Southeast Mediterranean Regions. *Plants (Basel)* 9, 2020

**De Mesquita ML, De Paula JE, Espindola LS, Soares LAL, Da Silva TMG, Camara CA, Da Silva TMG.** Protoflavanones from the Wood Stem of Salvertia convallariodora. *Nat Prod Commun* 12, 515-8, 2017

**Droissart V, Cribb PJ, Simo-Droissart M, Stévart T.** Taxonomy of Atlantic Central African orchids 2. A second species of the rare genus Distylodon (Orchidaceae, Angraecinae) collected in Cameroon. *PhytoKeys*, 27-34, 2014

**Efimenko TA, Shanenko EF, Mukhamedzhanova TG, Efremenkova OV, Nikolayev YA, Bilanenko EN, Gernet MV, Grishin AG, Serykh IN, Shevelev SV*, et al.*** Eurotium Cristatum Postfermentation of Fireweed and Apple Tree Leaf Herbal Teas. *Int J Food Sci* 2021, 6691428, 2021

**Fecka I, Kowalczyk A, Cisowski W.** Phenolic acids and depsides from some species of the Erodium genera. *Z Naturforsch C J Biosci* 56, 943-50, 2001

**Greiner S, Lehwark P, Bock R.** OrganellarGenomeDRAW (OGDRAW) version 1.3.1: expanded toolkit for the graphical visualization of organellar genomes. *Nucleic Acids Res* 47, W59-w64, 2019

**Guisinger MM, Kuehl JV, Boore JL, Jansen RK.** Extreme reconfiguration of plastid genomes in the angiosperm family Geraniaceae: rearrangements, repeats, and codon usage. *Mol Biol Evol* 28, 583-600, 2011

**Han A, Hwang JH, Lee SY.** Antimicrobial activities of Asian plant extracts against pathogenic and spoilage bacteria. *Food Sci Biotechnol* 32, 229-38, 2023

**Jin JJ, Yu WB, Yang JB, Song Y, dePamphilis CW, Yi TS, Li DZ.** GetOrganelle: a fast and versatile toolkit for accurate de novo assembly of organelle genomes. *Genome Biol* 21, 241, 2020

**Katoh K, Rozewicki J, Yamada KD.** MAFFT online service: multiple sequence alignment, interactive sequence choice and visualization. *Brief Bioinform* 20, 1160-6, 2019

**Katoh K, Misawa K, Kuma K, Miyata T.** MAFFT: a novel method for rapid multiple sequence alignment based on fast Fourier transform. *Nucleic Acids Res* 30, 3059-66, 2002

**Kong C, Pang X, Su Z, Liu Y.** Botany, ethnopharmacology, phytochemistry and pharmacology of Erodii Herba Geranii Herba-An review. *J Ethnopharmacol* 302, 115858, 2023

**Letunic I, Bork P.** Interactive Tree Of Life (iTOL) v5: an online tool for phylogenetic tree display and annotation. *Nucleic Acids Res* 49, W293-w6, 2021

**Levy M, Steiner EE, Levin DA.** Allozyme genetics in permanent translocation heterozygotes of the Oenothera biennis complex. *Biochem Genet* 13, 487-500, 1975

**Lewis SE, Searle SM, Harris N, Gibson M, Lyer V, Richter J, Wiel C, Bayraktaroglu L, Birney E, Crosby MA*, et al.*** Apollo: a sequence annotation editor. *Genome Biol* 3, Research0082, 2002

**Liu S, Ni Y, Li J, Zhang X, Yang H, Chen H, Liu C.** CPGView: A package for visualizing detailed chloroplast genome structures. *Mol Ecol Resour* 23, 694-704, 2023

**Minh BQ, Schmidt HA, Chernomor O, Schrempf D, Woodhams MD, von Haeseler A, Lanfear R.** IQ-TREE 2: New Models and Efficient Methods for Phylogenetic Inference in the Genomic Era. *Mol Biol Evol* 37, 1530-4, 2020

**Munekata PES, Alcántara C, Collado MC, Garcia-Perez JV, Saraiva JA, Lopes RP, Barba FJ, do Prado Silva L, Sant'Ana AS, Fierro EM*, et al.*** Ethnopharmacology, phytochemistry and biological activity of Erodium species: A review. *Food Res Int* 126, 108659, 2019

**Poinern GE, Le XT, Fawcett D.** Superhydrophobic nature of nanostructures on an indigenous Australian eucalyptus plant and its potential application. *Nanotechnol Sci Appl* 4, 113-21, 2011

**Raman G, Choi KS, Lee EM, Morden CW, Shim H, Kang JS, Yang TJ, Park S.** Extensive characterization of 28 complete chloroplast genomes of Hydrangea species: A perspective view of their organization and phylogenetic and evolutionary relationships. *Comput Struct Biotechnol J* 21, 5073-91, 2023

**Ruhlman TA, Zhang J, Blazier JC, Sabir JSM, Jansen RK.** Recombination-dependent replication and gene conversion homogenize repeat sequences and diversify plastid genome structure. *Am J Bot* 104, 559-72, 2017

**Schultz DJ, Suh MC, Ohlrogge JB.** Stearoyl-acyl carrier protein and unusual acyl-acyl carrier protein desaturase activities are differentially influenced by ferredoxin. *Plant Physiol* 124, 681-92, 2000

**Semenya SS, Maroyi A.** Medicinal plants used by the Bapedi traditional healers to treat diarrhoea in the Limpopo Province, South Africa. *J Ethnopharmacol* 144, 395-401, 2012

**Shi L, Chen H, Jiang M, Wang L, Wu X, Huang L, Liu C.** CPGAVAS2, an integrated plastome sequence annotator and analyzer. *Nucleic Acids Res* 47, W65-w73, 2019

**Wang YS, Huang R, Li NZ, Xu HY, Yang JH.** Triterpenes from Stachyurus himalaicus var. himalaicus Hook. f. et Thoms. ex Benth. *Molecules* 15, 2096-102, 2010

**Weng ML, Blazier JC, Govindu M, Jansen RK.** Reconstruction of the ancestral plastid genome in Geraniaceae reveals a correlation between genome rearrangements, repeats, and nucleotide substitution rates. *Mol Biol Evol* 31, 645-59, 2014

**Xu X, Huang H, Lin S, Zhou L, Yi Y, Lin E, Feng L, Zheng Y, Lin A, Yu L*, et al.*** Twelve newly assembled jasmine chloroplast genomes: unveiling genomic diversity, phylogenetic relationships and evolutionary patterns among Oleaceae and Jasminum species. *BMC Plant Biol* 24, 331, 2024

**Zhang D, Gao F, Jakovlić I, Zou H, Zhang J, Li WX, Wang GT.** PhyloSuite: An integrated and scalable desktop platform for streamlined molecular sequence data management and evolutionary phylogenetics studies. *Mol Ecol Resour* 20, 348-55, 2020

**Zhang YY, Li SH, Tian Z.** [Morphological and histological studies of the Chinese drug lao-guan-cao]. *Yao Xue Xue Bao* 30, 46-58, 1995

**Supplementary Figures**


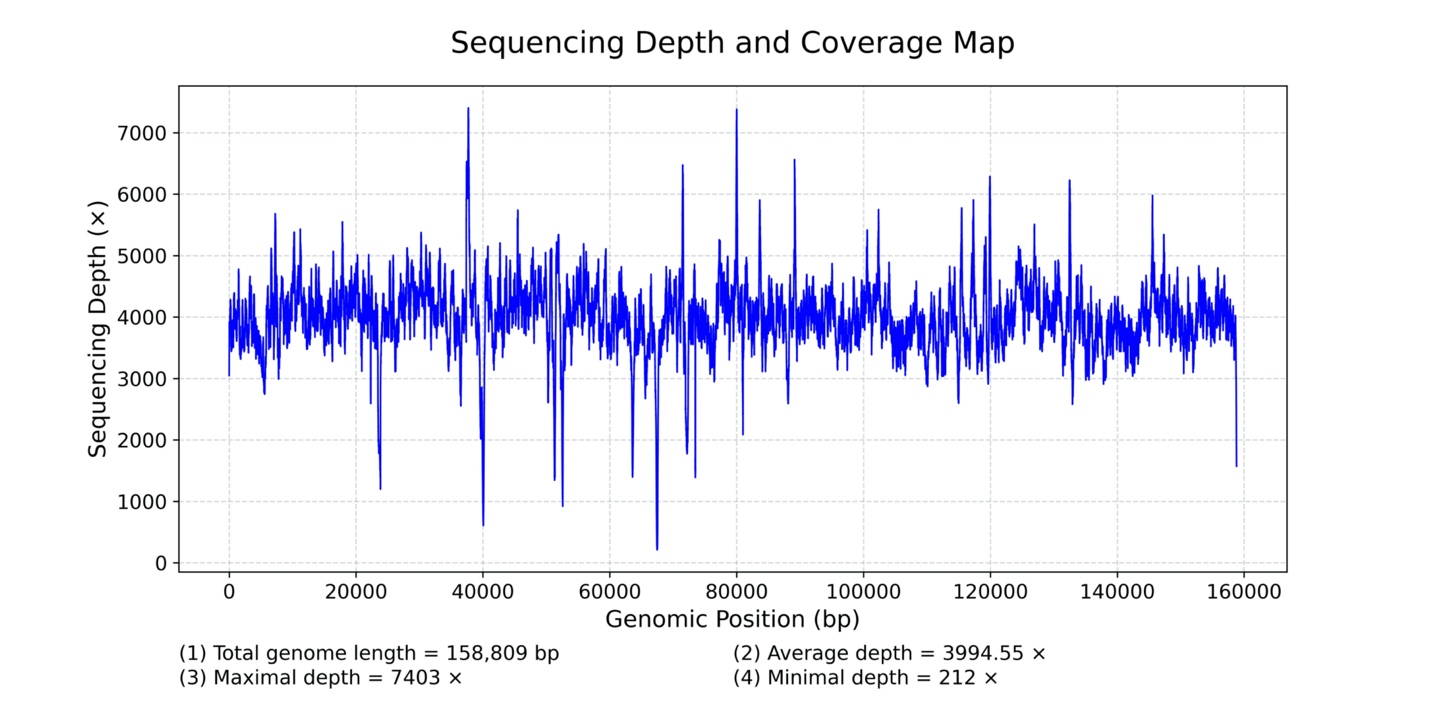


**Supplementary Figure 1.** Coverage depth distribution of the *E. stephanianum* cp genome.

**Supplementary Figure 2.** Structure of trans-splicing genes in the *E. stephanianum* cp genome.

**Supplementary Figure 3.** Structure of cis-splicing genes in the *E. stephanianum* cp genome.
