# Supplementary material for: The complete chloroplast genome of Erodium stephanianum (Geraniaceae)
Source: Mitochondrial DNA B Resour. 2024 Nov 12;9(11):1501–5. doi: 10.1080/23802359.2024.2419962 (PMC11559019; doi:10.1080/23802359.2024.2419962)
Supplement: es_Supplementary figures.docx [file TMDN_A_2419962_SM3108.docx]

**Supplementary Figures**


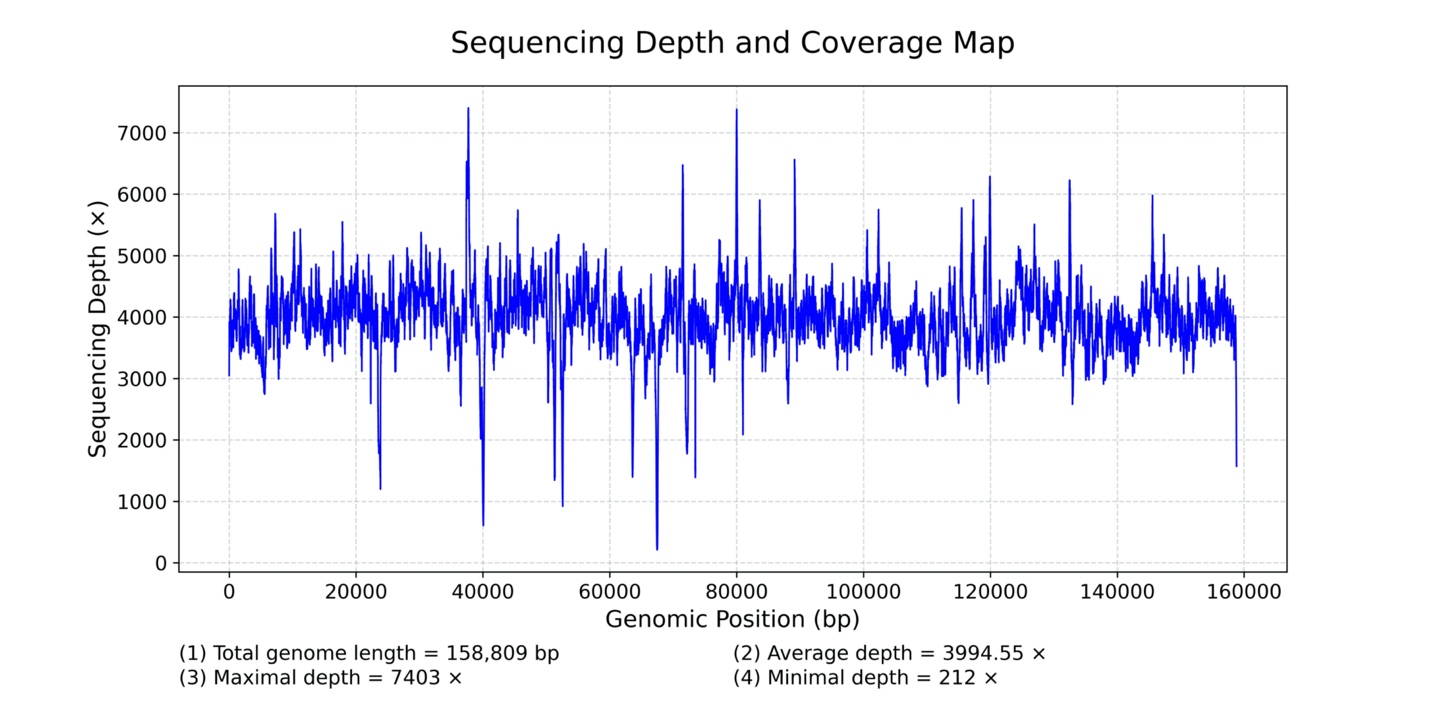


**Supplementary Figure 1.** Coverage depth distribution of the *E. stephanianum* cp genome.

**Supplementary Figure 2.** Structure of trans-splicing genes in the *E. stephanianum* cp genome.

**Supplementary Figure 3.** Structure of cis-splicing genes in the *E. stephanianum* cp genome.
